# Delusional Disorder, Erotomanic Type, Exacerbated by Social Media Use

**DOI:** 10.1155/2017/8652524

**Published:** 2017-03-07

**Authors:** Justin Faden, Jonathan Levin, Ronak Mistry, Jessica Wang

**Affiliations:** ^1^Lewis Katz School of Medicine, Temple University, 100 E. Lehigh Ave, Suite 305B, Philadelphia, PA 19125, USA; ^2^Rowan University School of Osteopathic Medicine, 42 E. Laurel Rd No. 1800, Stratford, NJ 08084, USA

## Abstract

Erotomania is an uncommon form of delusional disorder in which an individual has an unfounded belief that another is in love with him. Previous case reports have shown that social media networks may play a role in worsening delusional beliefs. We report the case of a 24-year-old male college student that utilized social media to stalk a female college student, resulting in his suspension from school and hospitalization. The student was diagnosed with delusional disorder, erotomanic type, and started on risperidone. He showed little improvement and was transferred to another facility. This is the first identified case of social media triggering or exacerbating delusional disorder. We recommend increasing education on the ramifications of sharing personal information on social media.

Erotomania is a form of delusional disorder in which an individual believes that another person, usually of higher status, is in love with him. It is a relatively rare condition, and while the incidence is unknown, the lifetime prevalence of delusional disorder is 0.2% [1]. Consequently, many psychiatrists do not encounter or may fail to recognize erotomania in their clinical practice. Although many theories exist for the etiology of delusional disorder, recent postulations have suggested that social media networks may play a role in enmeshing technology into the delusional systems of those predisposed to psychosis [2]. Social media networks are now ubiquitous aspects of modern society, and this implication cannot be overlooked. We present the case of a 24-year-old male with delusions of multiple women being romantically interested in him. He then engaged in stalking behavior via social media. Although social media has been linked to schizophrenia exacerbations, this appears to be the first identified case of exacerbated delusional disorder [2].

Mr. L is a 24-year-old male with no past family or personal psychiatric history who was brought to the emergency department by police for a psychiatric evaluation. The patient was discovered trespassing on local college property despite having been issued a No Contact Order following multiple complaints to the police and college administration by a female student. The student reported that he had been stalking her via Twitter and looking for her on the college campus. To substantiate her claims, she printed out a multitude of messages he sent her and gave these to the police and college administration. He proceeded to contact her several additional times, each time changing his Twitter username. He had been a student at the college; however, he was placed on suspension and ordered to remain off of campus property due to this behavior.

Interviewing revealed that Mr. L thought he was being unjustly persecuted and that these women did have romantic feelings for him. Although he primarily fixated on the female college student, secondary interests included another student and the Associate Dean that was responsible for reviewing the allegations made against him.

In the emergency department, the patient underwent a full laboratory workup consisting of a basic metabolic profile, complete blood count, rapid plasma reagin, and thyroid panel, all of which showed no significant findings. Urine drug screen was positive for cannabis. During the diagnostic interview, no mood symptoms were elicited, and no evidence of hallucinations, disorganized thoughts, or disorganized behavior was endorsed. The patient was diagnosed with delusional disorder, erotomanic type, due to the presence of nonbizarre delusions regarding multiple women being romantically interested in him despite no supporting evidence. Narcissistic traits were detected; however, his symptomatology was felt to be more consistent with a psychotic process. Second-generation antipsychotics are considered first-line pharmacologic treatment for delusional disorder, and previous reports have described success with olanzapine and risperidone, with those being the most frequently prescribed agents [3]. As the patient was naïve to neuroleptics, risperidone was chosen over olanzapine due to its more favorable metabolic profile. Risperidone was initiated and titrated to a dosage of 4 mg. Despite 14 days of treatment at that dosage, there was minimal improvement. A decision was made to transfer the patient to another hospital for further treatment after his college informed us that he had been calling both the Associate Dean and the female student. After transfer, he was lost to follow-up.

Delusional disorder remains a rare diagnosis, with the persecutory subtype being the most common and the erotomanic subtype being much rarer. The case of Mr. L is unique in that most cases of erotomania involve false beliefs about only one individual, and this is possibly the first reported case in which an individual with delusional disorder utilized social media as a means to facilitate his stalking behavior and augment his delusions. A previous report discusses the possibility of social media induced psychosis and how the use of symbolic language and interactive features on Twitter could contribute to or even worsen existing delusions [4]. Using Facebook and Twitter, Mr. L was able to constantly monitor his female classmates despite the No Contact Order.

Social networks have a presence in our daily lives and online communication can outweigh personal contact. Communication through social media can eliminate previous barriers that existed between an individual and the object of their delusions. Similarly, social media allows a form of anonymity or fictitiousness which previously did not exist. This case illustrates how any individual can use social media to fixate on, observe, and contact another person. Social media reduces privacy and increases accessibility for stalking behavior. Physicians and counselors should take this information into account when considering their patients' conditions, treatments, and restrictions. Swift action should be taken to reduce the likelihood of the behavior escalating. Similarly, parents and social network users should think closely about which personal details they choose to reveal on social networks, and we would recommend making this a mandatory feature of any school orientation. More research should be dedicated to exploring the interplay between social media and erotomanic delusions.
